# Essure^®^ removal in 10 steps

**DOI:** 10.4274/jtgga.galenos.2020.2020.0159

**Published:** 2021-02-24

**Authors:** Gautier Chene, Emanuele Cerruto, Erdogan Nohuz

**Affiliations:** 1 Department of Obstetrics and Gynecology, Hôpital Femme Mère Enfant, Lyon, France; 2 Department of Obstetrics and Gynecology Claude Bernard University, Lyon, France

**Keywords:** Essure, surgical technique, salpingectomy, laparoscopy

## Abstract

Many women request Essure^®^ removal because of possible side effects related to the device itself. Laparoscopic Essure^®^ removal in symptomatic women may be associated with improvement in quality of life. We aim to describe the surgical technique in ten steps in the accompanying video as the standardization of the laparoscopic Essure^®^ removal procedure could help to diminish the risk of fractures of the device with this easy and safe 10-step procedure.

## Introduction

Several studies have demonstrated an improvement of symptomatology and quality of life after removal of the Essure^®^ device in symptomatic patients (1,2). The pathophysiology of adverse effects related to the device may be explained by the release of heavy metals from a possible corrosion of the implant (3). Therefore, because there is a risk of fracture in up to 30% of cases (2), the implant should be removed completely and safely (4). Our aim was to give a step-by-step description of an easy surgical technique with a demonstrative video. 

### Surgery technique

**Figure 1:**
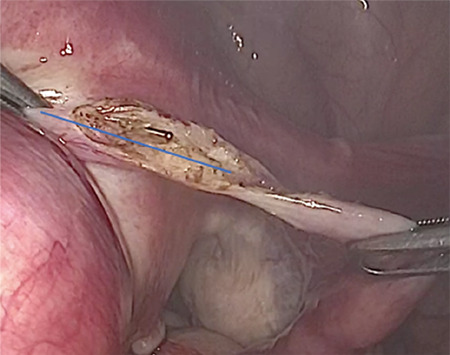
Longitudinal incision over the proximal fallopian tube towards the uterine horn

**Figure 2:**
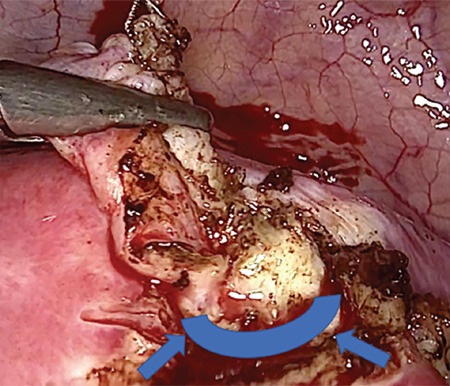
Circumferential incision on the 2/3 anterior portion of the fallopian tube

**Figure 3:**
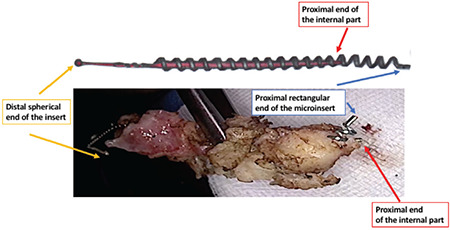
Inspection of the complete implant Essure^®^

This video clearly described the laparoscopic technique in 10 steps (Video 1): 1) pelvis exploration; 2) peritoneal cytology, for two reasons a) heavy metal analysis b) usually done in our department during prophylactic and opportunistic salpingectomy because of the potential tubal pathway for ovarian carcinogenesis (3,5); 3) longitudinal incision over the proximal fallopian tube towards the uterine horn (Figure 1); 4) circumferential incision around the interstitial tubal portion; 5) circumferential incision on the 2/3 anterior portion of the fallopian tube (Figure 2); 6) horizontal incision of the tube under the proximal rectangular end of the microinsert; 7) hemostasis of the uterine horn; 8) Essure^®^ removal under visual control; 9) Inspection and dissection of the Essure^®^ device on a surgical drape (Figure 3); 10) bilateral salpingectomy and other associated procedures, peritoneal washing and prevention of postsurgical adhesions. As compared with laparoscopic myomectomy, the small incision in the myometrium to perform this mini-cornuectomy should theoretically limit the risk of uterine rupture, if the patient wished to conceive via in vitro fertilization later. However further studies are required to confirm this retention of fertility (6).

## Conclusion

Since improvement of quality of life has been demonstrated after laparoscopic Essure^®^ removal in symptomatic women the standardization of the removal procedure could help to diminish the risk of fractures of the device.

**https://www.doi.org/10.4274/jtgga.galenos.2020.2020.0159.video1**
